# Influence of Chemical Enhancers and Iontophoresis on the In Vitro Transdermal Permeation of Propranolol: Evaluation by Dermatopharmacokinetics

**DOI:** 10.3390/pharmaceutics10040265

**Published:** 2018-12-07

**Authors:** María Aracely Calatayud-Pascual, María Sebastian-Morelló, Cristina Balaguer-Fernández, M. Begoña Delgado-Charro, Alicia López-Castellano, Virginia Merino

**Affiliations:** 1 Instituto de Ciencias Biomédicas, Departamento de Farmacia, Facultad de Ciencias de la Salud, Universidad Cardenal Herrera-CEU, CEU Universities, 46115 Alfara del Patriarca, Spain; maria.calatayud@uchceu.es (M.A.C.-P.); Maria.sebastian@uchceu.es (M.S.-M.); cbalaguer@uchceu.es (C.B.-F.); 2 Department of Pharmacy & Pharmacology, University of Bath, Claverton Down, Bath BA2 7AY, UK; B.Delgado-Charro@bath.ac.uk; 3 Instituto Interuniversitario de Investigación de Reconocimiento Molecular y Desarrollo Tecnológico (IDM), Universitat Politècnica de València, Universitat de València, 46100 Burjassot, Spain; Virginia.Merino@uv.es; 4 Departamento de Farmacia y Tecnología Farmacéutica y Parasitología, Universidad de València, Avda. Vicente Andrés Estellés sn, 46100 Burjassot, Spain

**Keywords:** iontophoresis, transdermal administration, dermatopharmacokinetics, propranolol, chemical enhancers

## Abstract

The aims of this study were to assess, in vitro, the possibility of administering propranolol transdermally and to evaluate the usefulness of the dermatopharmacokinetic (DPK) method in assessing the transport of drugs through stratum corneum, using propranolol as a model compound. Four chemical enhancers (decenoic and oleic acid, laurocapram, and R-(+)-limonene) and iontophoresis at two current densities, 0.25 and 0.5 mA/cm^2^ were tested. R-(+)-limonene, and iontophoresis at 0.5 mA/cm^2^ were proven to be the most efficient in increasing propranolol transdermal flux, both doubled the original propranolol transdermal flux. Iontophoresis was demonstrated to be superior than the chemical enhancer because it allowed faster delivery of the drug. The DPK method was sufficiently sensitive to detect subtle vehicle-induced effects on the skin permeation of propranolol. The shorter duration of these experiments and their ability to provide mechanistic information about partition between vehicle and skin and diffusivity through skin place them as practical and potentially insightful approach to quantify and, ultimately, optimize topical bioavailability.

## 1. Introduction

Propranolol is a widely used non-selective beta-adrenergic receptor blocker used to treat hypertension, coronary diseases (angina pectoris, arrhythmias), anxiety disorders, tremors, prevention of migraine and bleeding esophageal varices [1]. Moreover, propranolol is used as the first-line treatment in essential tremor [2], this drug has emerged as front-line therapy for infantile hemangiomas [3] and it has also been proposed as a treatment for tremor in dystonia [2]. It was recently shown to display anticancer properties because it synergizes with certain drugs [4].

Propranolol is a quite lipophilic drug (log *P*_oct_ = 3.03). It is administered intravenously and orally as tablets and although well orally absorbed, the mean absolute bioavailability is 30–35%. Pharmacokinetics parameters following a single oral dose (160 mg) are: maximal plasma concentrations = 28 ng/mL, apparent distribution volume = 3.6–4.5 L/kg, terminal elimination half time (*t*_max_) = 2 to 6 h and its clearance approximately 12 mL/min/kg [5,6].

Propranolol pharmacokinetic parameters differ between adolescents and adults, and its oral bioavailabity can vary up to 20 times from one individual to another, which requires multiple dose increases in some patients [5].

Due to its low bioavailability after oral dosing, that it is also dependent on food intake, inconveniences related to parenteral dosing and pharmacokinetic properties, as short terminal elimination half time, the exploitation of an alternative route of propranolol delivery, such as transdermal administration, could be of benefit. In this context, a transdermal delivery system of propranolol could be a new interesting pharmaceutical form.

Skin offers the possibility of acting as an administration route for the systemic delivery of therapeutic agents. Physicochemical properties such as the partition coefficient, the molecular weight and the solubility of a molecule determine primarily its skin transport. Skin diffusion is mainly controlled by its outermost layer, the SC, the properties of which may be manipulated by applying chemical penetration enhancers to the skin [7,8]. In addition, other methodologies have been proposed to enhance transdermal drug absorption. Among them iontophoresis, a non-invasive technique which acts principally on the drug rather than on the skin itself, is the most commonly used physical enhancement method used to promote skin transport. Iontophoresis consists on the application of a low-level electrical currents (with a current density <0.5 mA/cm^2^) onto the skin [9]. The application of an electric potential across the skin generates a flux of ions called electrorepulsion or electromigration. This ion flux represents the main mechanism of transport during iontophoresis. A second mechanism of transport is based on the electroosmotic or convective flow that occurs in the “anode-to-cathode” direction in physiological conditions and it is due to the net-electrical charge of the skin [9,10,11,12,13,14,15].

The dermatopharmacokinetic (DPK), or tape-stripping, method is a valuable tool to assess the rate and extent of topical drug absorption into the SC or local bioavailability. Sampling the SC using sequential applications of adhesive tape is minimally invasive. This technique has demonstrated its usefulness to evaluate SC cohesion in vivo by quantifying the amount of SC removed [16]. Moreover, DPK data provide information about drug kinetics in the SC by measuring the drug present in this layer at specific times post administration. For this reason, DPK has been used to assess differences between formulations and as a surrogate method to predict the bioavailability and assess the bioequivalence of topical products in a non-invasive methodology [17,18,19].

The present work aimed to investigate whether propranolol can be administered transdermally in an efficient manner using either chemical and physical enhancement methods. To do this, we have conducted a series of in vitro diffusion experiments to identify potential components of a transdermal delivery system of propranolol. Four chemicals, decenoic acid, oleic acid, laurocapram, and R-(+)-limonene, all with known efficacy as transdermal absorption enhancers, were selected [20]. In addition, the effect of iontophoresis (0.25 and 0.5 mA/cm^2^) on the transdermal delivery of propranolol were also investigated.

A second aim of this research was to evaluate the usefulness of the DPK method in assessing the transport of drugs through the SC, using propranolol as a model compound. To this end, tape-striping was used to determine the concentration profile of propranolol across the SC following different experimental conditions. Next, the data were interpreted using a straightforward diffusion-partition transport model to elucidate mechanistic information that might aid in designing more efficient methods to deliver drugs across and in the skin.

## 2. Materials and Methods

### 2.1. Materials

Propranolol hydrochloride certified standard (98.9% *w*/*w*) (Eur. Ph. Monograph 0568) and decenoic acid were provided by Sigma-Aldrich (Madrid, Spain). R-(+)-limonene was purchased from Fluka Chemie (Buchs, Switzerland). Oleic acid, ethanol (absolute), acetonitrile, ammonium *di*-hydrogen phosphate (96–102%, *w*/*w*) and NaCl were obtained from Análisis Vínicos, S.L. (Tomelloso, Spain). Orto-phosphoric acid (85–88%, *v*/*v*) was purchased from Panreac Química S.A. (Barcelona, Spain). HEPES (*N*-[2-Hydroxiethyl] piperazine-*N*′-[2-ethanesulfonic acid]), NaOH, HCl analytical grade and silver chloride (99%), silver and platinum wire 1 mm (both 99.9%), used to make the Ag/AgCl electrodes, were obtained from Sigma-Aldrich Co. (St. Louis, MO, USA). Laurocapram (1-dodecyl-azacycloheptan-2-one) was obtained from Netqem (Durham, NC, USA). All compounds were of HPLC grade. Ultrapure water was obtained with a Barnstead NANOpure system (Barnstead International, Boston, MA, USA) and it was used in the preparation of solutions.

For preparing the salt bridges employed in the iontophoretic studies, agarose ultrapure electrophoresis grade and silicone tube with 3.5 mm internal diameter were purchased from Sigma-Aldrich Co (St. Louis, MO, USA) and Levantina Laboratorios S.L. (Valencia, Spain), respectively. The electrical current applied on the skin was provided by a Kepco BHK-MG-0-2000 V power supply (Kepco, Inc., Flushing, NY, USA).

Porcine skin was obtained, for the various experiments here described, following animal sacrifice. Porcine ear skin was used for long-term percutaneous diffusion studies [21,22] whereas DPK tests used skin from the dorsal and abdominal areas [23,24]. Pig ears were generously donated by the Faculty of Medicine, University of Valencia (Valencia, Spain). Skin from the outer side was excised from the ear using a surgical blade. Afterwards it was dermatomed using an Aesculap-Wagner dermatome C. GA 176 (B. Braun surgical S.A., Barcelona, Spain) to a nominal thickness of 600 µm. The dorsal and abdominal skin was sourced from a local abbatoir, and was gently washed under cold running water post-sacrifice at a local abattoir. Afterwards it was dermatomed to a nominal thickness of 750 µm (Zimmer^TM^ Electric Dermatome, Dover, NH, USA). The tissue samples obtained were wrapped individually in Parafilm^TM^ and stored for no more than three months at −80 °C until use.

The project (AP2007-03456) was approved by the Committee of the Universidad CEU Cardenal Herrera (May 2007).

### 2.2. Diffusion Experiments

Transdermal permeation of propranolol was investigated during 30 h. Assays were performed employing vertical Franz-type diffusion cells (DISA, Milan, Italy) with a diffusion area of 0.567 cm^2^.

The dermatomed pig ear skin was defrosted and then placed between both sides of the cell so that the SC faced the donor compartment.

The receptor compartment of the diffusion cells (4.2 mL volume) was filled with a saline buffer (NaCl 150 mM, HEPES 20 mM, pH 7.4) and stirred by a rotating Teflon-coated magnet to prevent possible boundary layer effects. The lower, receptor compartment of the assembled cells was introduced in a bath thermostated at 37 °C (36.96 ± 0.14 °C), so the temperature of the skin was maintained at 32 °C (32.3 ± 1.2 °C).

A first series of experiments investigated whether ethanol or the potential enhancers promoted the percutaneous permeation of propranolol with respect to the control. For this, the skin was treated for 12 h with either 200 µL of a saline-buffered solution at pH 7.4 (control), ethanol (vehicle control), or a 5% (*w*/*w*) chemical enhancer [decenoic acid, or oleic acid, or R-(+)-limonene or laurocapram (Azone^®^)] solution in ethanol, respectively [20]. Following this treatment, the solutions were removed from the donor compartment and 1 mL of propranolol solution (20.3 µM) in saline buffer (NaCl 25 mM, HEPES 20 mM, pH 7.4) was placed in the donor compartment [25]. Then, the passive permeation of the drug was followed for 30 h.

Iontophoresis assays were conducted to investigate the effect of application of current on the skin permeation of propranolol. These studies involved an 8 h iontophoresis phase followed by a 22 h post-iontophoresis passive stage. Prior to iontophoresis no pre-treatment of the skin took place. Constant currents of 0.142 and 0.842 mA, resulting in current densities of 0.25 and 0.5 mA/cm^2^, were delivered using Ag/AgCl electrodes connected to a Kepco BHK-MG 0–2000 V power supply.

The electrode anode chamber was separated from the donor chamber using a salt bridge made with 3% agar and 0.1 M NaCl in water. For this, the heated mixture was introduced in silicon tubes and allowed to dry at room temperature [26]. The receptor cathodal solution was 4.2 mL of 150 mM NaCl buffered to pH 7.4 with HEPES 20 mM and the donor solution consisted in 20.3 µM propranolol in water.

In all the experiments (passive controls, chemical enhancer assays and iontophoretic experiments) 200 µL samples were manually taken from the receptor chamber at predetermined time intervals. The sample volume taken was immediately replaced with fresh buffer pH 7.4.

At the end of the in vitro transdermal permeation experiments, the amount of propranolol retained in the skin was extracted by shaking the tissue during 12 h in 3 mL of the saline buffer (pH 7.4). Drug recovery was (91.3 ± 4.3) % (*n* = 10). After the extraction procedure, a sample from the extract was filtered by Minisart^®^ filter with 0.45 µm diameter (Sartorious AG, Hannover, Germany) and analysed for propranolol.

### 2.3. Data Analysis of In Vitro Diffusion and Iontophoretic Experiments

The data corresponding to the permeation of propranolol in the different passive conditions was analysed as it follows. First, plots of the accumulated amount of the drug (µg/cm^2^) in the receptor against time (hours) were constructed. And next, the accumulated amount versus time was fitted using Equation (1) [27]:

(1) $$Q=\left(KH\right)C_{veh}\left[\frac{D}{H^{2}}t-\frac{1}{6}-\frac{2}{\pi^{2}}\overset{\infty }{\underset{n=1}{\sum }}\frac{{\left(-1\right)}^{n}}{n^{2}}\exp\left(\frac{-Dn^{2}\pi^{2}t}{H^{2}}\right)\right]\;,$$

The boundary conditions necessary are: (a) the SC contains no drug at *t* = 0; (b) the drug concentration at the skin surface is constant (infinite dose conditions); and (c) the viable epidermis at the lower surface of the SC provides perfect sink conditions for the drug. *Q* is the cumulative amount of drug permeated per unit area at time *t*, *C_veh_* is the concentration of the drug in the donor vehicle, *K* is the SC/vehicle partition coefficient, *D* the diffusion coefficient, and *H* the diffusion path length.

In long term assays performed using dermatomed skin the value of D corresponds to the mean diffusion coefficient through skin, since for this calculation it is assumed that skin behaves as a homogeneous membrane.

The fitting was performed using WinNonlin Professional 5.0.1 Software (Pharsight Corp., Mountain View, St. Louis, MO, USA).

This procedure led to the determination of the drug’s partition parameter (*KH*) and diffusivity (*D*/*H*^2^). The permeability coefficient (*K_p_*) was then calculated as the product of *KH* and *D*/*H*^2^:

(2) $$K_{p}=KH\cdot \frac{D}{H^{2}},$$

The lag time of diffusion (*t_o_*) was determined from the expression:

(3) $$t_{o}=\frac{H^{2}}{6D},$$

And the steady-state flux (*J*) was predicted from:

(4) $$J=\;K_{p}\cdot C_{veh},$$

The values of *J*, *KH* and *D*/*H*^2^ resulting from the above analysis and the experimentally measured amount of propranolol recovered from the skin at the end of the experiments were compared using the one-way ANOVA test, followed by the post hoc tests Scheffé or T3 Dunnet.

In the iontophoresis assays, the steady state transdermal flux (*J*) was estimated from the slope of the linear region of the accumulated amounts of propranolol against time.

When statistically differences were detected by means of the ANOVA test (*p* <0.05) the permeation enhancing activity, expressed as enhancement ratio of flux (ER_flux_), was calculated as the ratio of the flux value obtained with the chemical enhancers or iontophoresis with respect to that found with the passive control.

### 2.4. Tape-Stripping Experiments

After the preceding studies, the in vitro distribution profiles of propranolol through the SC as well as the DPK parameters deriving were compared to better understand the transdermal permeation of the beta-blocker in the different conditions studied. The diffusion experiments were similar to those described above with the following exceptions: (a) Horizontal diffusion cells with a diffusion area of 3.8 cm^2^ were used; (b) The studies with the chemical enhancers (ethanol, laurocapram, R-(+)-limonene and oleic acid) were conducted at 32.2 ± 0.5 °C, *n* = 5 and the iontophoresis experiments were carried out at room temperature (24.0 ± 0.2 °C); (c) Finally, the experiments lasted 3 h after which, a single sample from the receptor chamber was taken and the tape-stripping procedure was done. In these studies, the pre-treatment with enhancers was the same than that with longer studies (that is; 12 h application of chemical enhancers in passive studies and no pre-treatment for iontophoresis studies).

Layers of SC were progressively removed by consecutively applying and removing tapes (Scotch Book Tape, 3M, St. Paul, MN, USA). To ensure that the tapes removed the SC from the same location, a 2 cm diameter template was fixed to the skin. The adhesive tapes (2.5 × 2.5 cm) were applied to this template, pressed down with a constant pressure (140 g/cm^2^) using a weighted roller (6 cm width) and then removed. Up to 20 strips were taken from each treated site (area diffusion), such that the SC was completely removed. To evaluate the skin barrier function, transepidermal water loss (TEWL) measurements were performed (AquaFlux V4.7, Biox Systems Ltd., London, UK) during the stripping procedure, which was stopped if TEWL reached 60 g/m^2^h. Each tape was carefully weighted before and after stripping on a 0.1 µg precision balance (Sartorious SE2-F, Epsom, UK) to determine the mass and thickness of the SC layer removed.

Finally, each tape was placed into a 2 mL vial, and was extracted with 1 mL of 70:30 (*v*/*v*) acetonitrile/water by shaking overnight. The amount of propranolol extracted was subsequently analysed by high-performance liquid chromatography (HPLC) (see method below). Validation of the extraction process involved spiking tape-stripped samples of untreated SC with 50 µL of a solution of known drug concentration (56.3 µM of propranolol). Drug recovery was (85.4 ± 1.5) % (*n* = 5).

The amount of propranolol on each strip could then be converted to a concentration within that removed layer of the SC.

To calculate the total thickness of the SC, the same tape-stripping procedure was performed at an adjacent, untreated skin area with measurements of TEWL taken after each tape-strip. Pre-weighted tapes were used, which were re-weighed after a SC layer had been detached to assess the amount of SC removed. From this mass, and knowing the stripping area and the density of the SC (1 g/cm^3^), it was possible to calculate the total thickness of the SC from the *x*-axis intercept of a graph of 1/TEWL versus the cumulative thickness of the SC removed [28,29].

In this way, the drug concentration profile across the SC could be displayed in a consistent fashion for all condition studied, as a function of relative position (or depth) into the skin barrier.

### 2.5. Analysis of the SC Distribution Profile

In order to study the distribution of propranolol in the SC, the SC concentrations (*C_x_*) *versus* normalized depth (*x*/*H*) profiles of propranolol, following passive experiments with and without chemical enhancers, were fitted to the appropriate solution of Fick’s second law of diffusion [30]:

(5) $$C_{x}=KC_{v}\left[1-\frac{x}{H}-\frac{2}{\pi }\overset{\infty }{\underset{n=1}{\sum }}\frac{1}{n}\sin\left(n\pi \frac{x}{H}\right)exp\left(-\frac{D}{H^{2}}n^{2}\pi^{2}t\right)\right],$$

From the best fit of the experimental values to Equation (5), the SC-vehicle partition coefficient (*K*) and the diffusivity parameter (*D*/*H*^2^) across the SC of path-length *H* were obtained.

The area under the curve of the propranolol SC concentrations (*C_x_*) versus normalized depth (*x*/*H*) was calculated using the trapezoidal method. In addition, the total amount (*A*) of propranolol in the SC was calculated with the following equation:

(6) A= AUC⋅H,

The parameters *K*, *D*/*H*^2^, *AUC* and *A* were used to compare permeation kinetics of the drug in the SC in the different conditions tested. In the case of the iontophoretic experiments only AUC and *A* were calculated.

Results were compared using the one-way ANOVA. To determine between which groups there were statistically significant differences, the Bonferroni’s multiple comparisons test was performed.

### 2.6. Analytical Methods

The amount of propranolol in all samples was quantified by high performance liquid chromatography (HPLC) using a Waters liquid chromatograph (Waters 600 Controller and Pump) which included a diode-array detector (Waters 996 Photodiode Array Detector) (Waters, Barcelona, Spain), set to 291 nm and an analytical Kromasil^®^ C_18_ column (250 × 4 mm, 5 µm) (Análisis Vínicos, Tomelloso, Spain). A mixture of monobasic ammonium phosphate water solution (0.05 M, pH 3.7)-acetonitrile (69:40, *v*/*v*) was used as mobile phase, at a flow rate of 1 mL/min injection volume was 50 µL. The method had been previously validated [31]. The limits of detection and of quantification were 0.171 and 0.115 µM, respectively.

## 3. Results and Discussion

**Figure 1:**
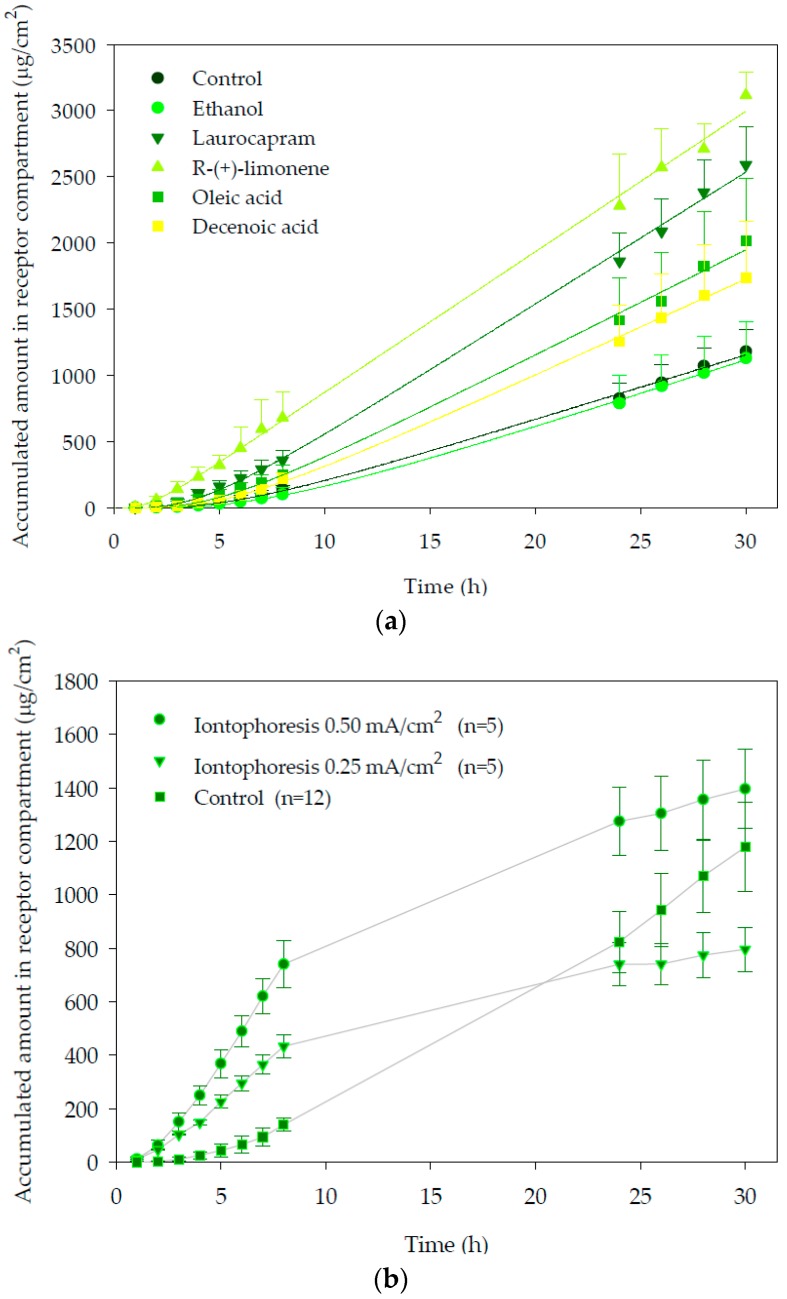
Accumulated amounts of propranolol (µg/cm^2^) in the receptor compartment versus time (h), measured during in vitro transdermal experiment: (**a**) passive diffusion with chemical enhancers; (**b**) iontophoresis. Error bars show standard deviation (SD), *n* ≥ 6. Current was switched off at 8 h.

The cumulative amounts of propranolol in the receptor compartment as a function of time after chemical enhancers pre-treatment are plotted in Figure 1a. As can be seen, pre-treatment of skin with R-(+)-limonene increased significantly the penetration of the drug through the skin and provided the largest cumulative amount of propranolol in the receptor compartment. With laurocapram and oleic acid, a moderate increment of the amount of propranolol permeated through the skin and ethanol (vehicle control) was observed. Finally, decenoic acid did not modify propranolol drug delivery to the receptor with respect to the observed with the control.

Table 1 summarizes the steady state flux (*J*), the amount of drug extracted from the skin at the end of the experiments, and the enhancement ratio of steady state flux (ER_flux_) calculated for all the strategies applied. The permeation parameters *KH* and *D*/*H*^2^ were calculated for passive experiments with and without enhancers, by fitting the experimental data to a solution of second Fick’s law (Equation (1)) to provide a mechanist insight (see Table 1). The parameter *KH* gives indications as to the SC/vehicle partitioning of the molecule, while *D*/*H^2^* represents the diffusive parameter across the skin.

As can be seen in Table 1, the transdermal flux value at passive diffusion was 48.9 ± 1.3 µg/cm^2^h and the amount of propranolol retained in the skin after 30 h was 302 ± 81 µg/cm^2^ (mean ± SD; *n* = 12). Propranolol is a weak base (pKa = 9.45), lipophilic and small molecule (log P_oct_ = 3.03 and MW = 295.8 g/mol) therefore, as expected, propranolol has a reasonable transdermal flux [32]. The results presented in the Table 1 show that the value for the parameter *KH* is 0.31 ± 0.03 cm, approximately 3.5 times greater than other less lipophilic beta-blockers assayed in the same passive diffusion experimental condition [33].

The control vehicle (ethanol) provided a transdermal flux (51.0 ± 1.2 µg/cm^2^h) that did not differ significantly from that observed with the control.

As shown in Table 1, pre-treatment with laurocapram, R-(+)-limonene and oleic acid led to a statistically significant increment of the transdermal flux of propranolol (*p* < 0.05), providing a flux value of 99.7 ± 2.0, 102 ± 2 and 79.6 ± 2.4 µg/cm^2^h, respectively.

The chemical enhancers evaluated in this study were chosen as representatives of different mechanisms of action. Laurocapram produces a fluidifying action on the skin lipids reducing the diffusional resistance of the stratum corneum and also affects the hydration of the SC [34]. Terpenes as R-(+)-limonene, improve drug partitioning into the SC by modifying its solubilizing properties [35,36,37,38,39]. Fatty acids, as oleic and decenoic acid, also increase partitioning of drugs into the skin because they disrupt molecular packaging and degree of hydration of the SC [40,41].

Table 1 shows that no differences were found between the experiments conducted with the buffer and the ethanol controls. For decenoic acid, a small but significant increase was observed for the diffusivity parameter although this was insufficient to enhance significantly delivery of the drug. On the other hand, laurocapram, limonene and oleic acid experiments provided significantly higher propranolol fluxes than the two controls, which in all cases resulted from a significant increase in the diffusivity parameter (with respect to both controls); from an increase in the partitioning with respect to the buffer control by the three enhancers and in the partitioning parameter with respect to ethanol in the case of oleic acid. Propranolol is positively charged at pH 7.4 whereas oleic acid has a negative charge; potentially an ionic interaction between both could contribute to the retention of the drug into the skin. However, decenoic acid also negatively charged (−1) at pH 7.4 did not cause similar retention of the drug into the skin, so there is no clear support for this hypothesis. Consequently, the higher amount of the drug accumulated on the skin in the experiment with oleic acid was considered mainly due to an increase in the partitioning parameter.

The chemical enhancers assayed in this study differ in their lipophilicity. The highest transdermal flux of propranolol was obtained with R-(+)-limonene, a compound with an estimated log *P*_oct_ value of 4.23. More lipophilic enhancers (laurocapram, predicted log *P*_oct_ value of 5.9; oleic acid, predicted log *P*_oct_ value of 6.5) and the more hydrophilic molecule (decenoic acid, log *P*_oct_ value of 3.8) (Pubchem data) were less efficient in promoting the transdermal delivery of propanolol. The *KH* parameter value tended to increase with the lipophilicity of the chemical enhancer (Table 1), although only oleic acid increased *KH* significantly with respect to the ethanol control. Such trend was not observed for the *D*/*H^2^* parameter. All enhancers, except decenoic acid, provided higher values of *D*/*H^2^* than the ethanol control. The maximum *D*/*H^2^* was observed with the terpene, consistently with the maximum effect observed in drug permeation.

Previously, an optimum value in the lipophilicity of the substance used as chemical percutaneous enhancer has been reported [20], suggesting that the chemical enhancer lipophilicity required for the best effect varies depending on the lipophilicity of the drug studied. The data here presented support these previous findings but also, through the determination of *KH* and *D*/*H^2^* parameters provide some insight into the mechanism of actions involved.

With respect to iontophoresis, Figure 1b. shows the amounts of propranolol accumulated in the receptor compartment as a function of time. It can be observed that during iontophoresis application the permeation vs. time profile was linear from the beginning of the assays. Pseudo steady-state transdermal fluxes (*J*) were estimated from the slope of the linear region and are shown in Table 1. Statistical analysis of flux and total amount accumulated in receptor chamber, during the first 8 h showed that application of a current density of 0.5 mA/cm^2^ significantly increased (*p* < 0.05) (2.12-fold) propranolol delivery with respect to the passive control and to the 0.25 mA/cm^2^ iontophoretic condition (1.75-fold). This is in agreement with previous reports that support that iontophoretic fluxes are proportional to the applied current intensity [11,25,42].

When current was switched off, the cumulative curve tended progressively to flatten over time, indicating the much smaller passive diffusion contribution to the total flux measured during iontophoresis.

The transport numbers for propranolol in iontophoresis experiments across pig ear were calculated as described previously [43]. These values (mean ± SD) were (1.30 ± 0.16) % and (1.18 ± 0.16) % for 0.25 and 0.5 mA/cm^2^, respectively, indicating that the drug transports less than 1.5% of the total charge delivered across the skin in all cases. This value depends on the competition for charge carrying by ions present in both, in the donor and the subdermal solution. Whilst subdermal competition cannot be avoided, it would be possible to optimize the efficiency of transport by minimizing the presence of competing cations present in the donor solution either originally or originating from the salt-bridge. Whilst further optimization is possible, the current value is not further from that obtained with propanolol in the absence of competing co-ions reported [44] and consistent with the quick decrease in the efficiency of transport numbers with molecular weight [43,44].

The transdermal fluxes provided by iontophoresis at 0.5 mA/cm^2^ and the enhancer R-(+)-limonene were very similar. However, another important aspect in transdermal absorption is the lag time, a value related with the time required to reach the steady state diffusion. In the case of iontophoresis experiments, an “apparent” lag time was estimated as the intercept of linear regression into x axis, for comparison purposes only, as this parameter lacks any mechanistic information in this case. Table 1 shows the lag time measured in the passive experiments, indicating the delay until measurable drug concentrations have reached the receptor solution. The chemical enhancers did not decrease lag time compared to the passive control with the exception of R-(+)-limonene that reduces its value to 1.80 ± 0.27 h. The “apparent” lag time measured in iontophoretic experiments cannot be interpreted with passive diffusion theory; however, from a practical point of view, it is obvious that propranolol reaches the subdermal compartment much faster when iontophoresis is employed which would lead to faster attainment of relevant plasma levels.

Taking into account propranolol systemic clearance, it can be considered that R-(+)-limonene and iontophoresis would enable plasma concentrations similar to those reached after oral administration (approximately 28 ng/L) using a transdermal system of a reasonable size (around 15 cm^2^). These results confirm the possibility of administering propranolol through the skin.

**Figure 2:**
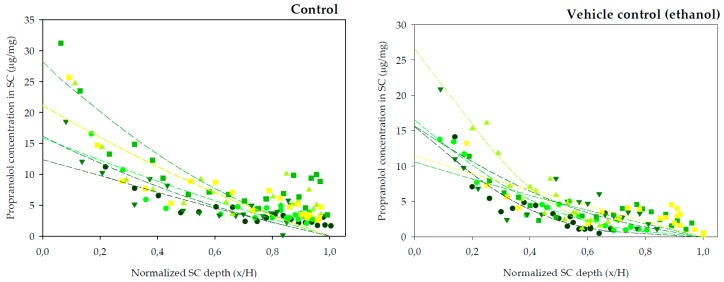

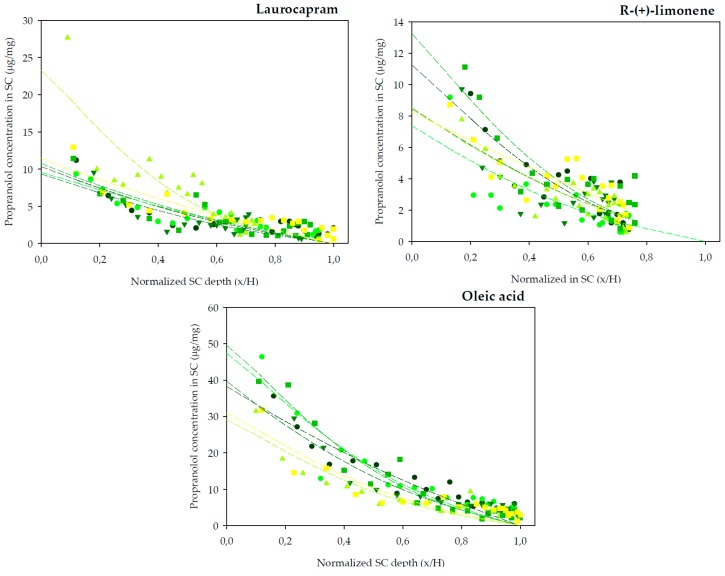
SC distribution profiles of propranolol across SC following in vitro experiments following a (*n* = 6). The lines of best fit of Equation (5) through the individual experimental data points are shown.

**Figure 3:**
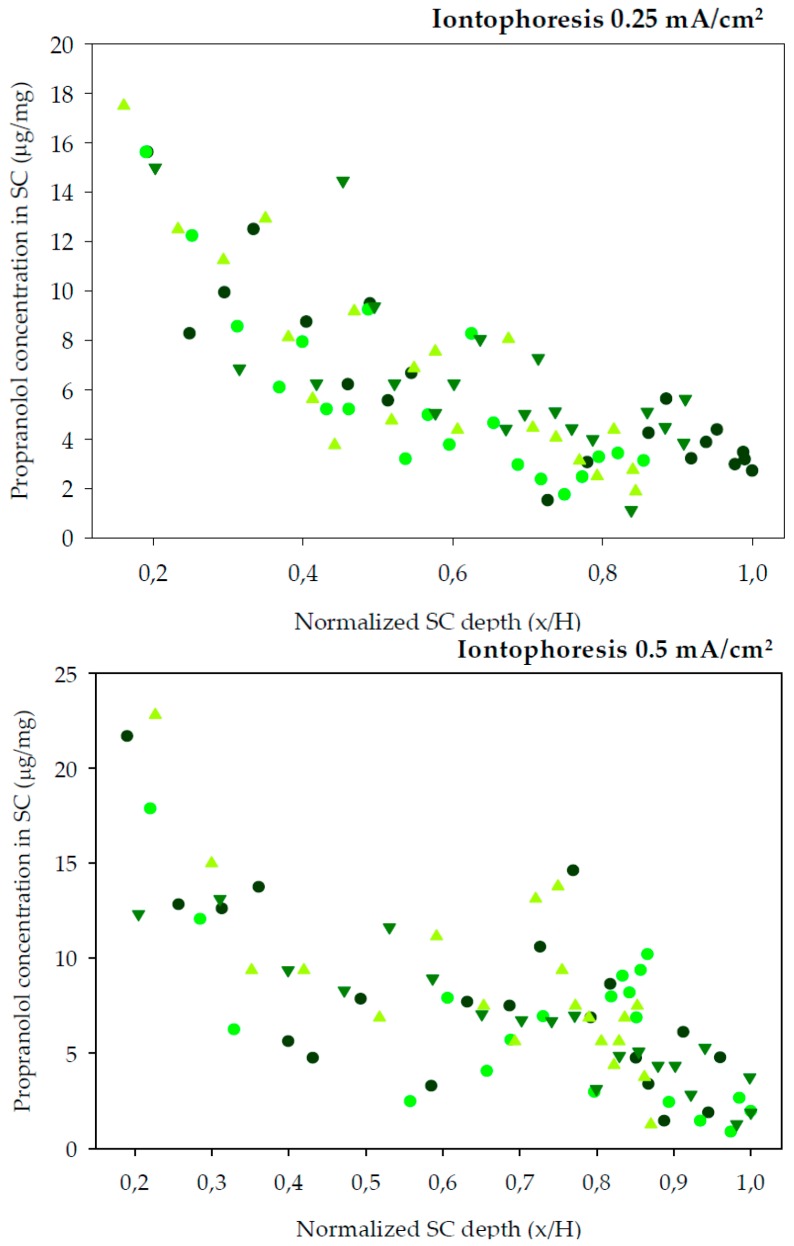
SC distribution profiles of propranolol across pig SC in vitro following a 3 h iontophoresis application (*n* = 4).

Finally, tape-striping was used to determine the concentration profile of propranolol across the SC following different experimental conditions. The experimental design of the tape-stripping and the previous in vitro permeation tests primarily differed in the application time. This was done because Equation (5) describes the SC distribution profile of a drug before attainment of the diffusional steady-state (~2.7 *t*_o_) [45]. The previous experiments indicated a lag time of approximately 2 h for R-(+)-limonene, so a range of preliminary experiments lasting between 2 and 4 h were performed. The longest, 4 h experiments, led to practically linear profiles of the drug across the SC, thus close to the steady state condition, whereas the shortest tests (2 and 2.5 h) rendered very low concentrations of the drug in the SC, that were difficult to quantify. Thus, the final experiments involved 3 h of exposure considered best for the calculation of the dermatopharmacokinetic parameters. Other differences regarding the diffusion area of the assays and temperature at which iontophoresis experiments were carried out were imposed by the facilities available at the different locations at which the experiments took place. Nevertheless, the changes are not considered relevant: on one side skin surface is computed in the calculations; on the other side, iontophoretic transport is primarily controlled by the intensity of current applied which was constant, temperature has a minor effect on drug transport under these circumstances. The SC distribution profiles of propranolol after a 3 h diffusion experiments are plotted in Figure 2 (chemical enhancers) and Figure 3 (iontophoresis).

The amount of SC sampled by tapes is affected by different factors, for example the cohesion between corneocytes is modified by potent enhancers [46]. Another factor is the inter-individual variability in total SC thickness. Thus, the amount of SC sampled is not linearly proportional to the number of tape-strips removed. By normalizing the depth of SC sampled with respect to the total SC depth for each skin sample, comparisons between the different conditions are facilitated. Therefore, the distribution profiles in Figure 2 and Figure 3 have been normalized with respect to the average SC thickness, *H*, 8.69 ± 0.62 μm (mean ± SD).

The best fits of Equation (5) to the data are drawn through the individual points in Figure 2. The deduced values of *K* and *D*/*H^2^* from these measurements are shown in Table 2. *AUC* and the total amount of drug in the SC (*A*) were calculated to compare delivery with chemical enhancers and iontophoresis (Table 2).

Table 2 indicates that after 3 h of permeation, only the oleic acid delivers more drug to the stratum corneum than the control, an effect due to increased partitioning. Whilst limonene seems to increase the diffusivity parameter, this is not sufficient to provide enhanced delivery of the drug, perhaps due to the reduced (albeit not significantly) partitioning. On the other hand, the highest partition coefficients (*K*) SC/vehicle were obtained with the buffer control and the oleic acid experiments; the other experimental conditions reduced the value of this parameter. DPK results in Table 1 and Table 2 agree in identifying oleic acid as an enhancer that most increased the partitioning of propranolol and limonene as the enhancer that most increased the diffusivity parameter of the drug. Thus, the two experimental approaches led to same qualitative conclusions regarding the primary mechanism of action of the enhancers. Quantitatively, however, there are differences between the values observed explained by different length of the assays, and primarily, by the different skin compartments interrogated with the two methods: the DPK approach assess primarily the kinetics of the drug in the SC whereas the permeation studies assessed the kinetics of the drug in the receptor solution, a step that can also be affected by the diffusion of the drug through the viable tissue.

The total amount of propranolol in the SC can be observed in Table 2. As can be seen, only oleic acid and iontophoresis at the higher current density (0.5 mA/cm^2^) statistically increased (*p* <0.05) the total amount of propranolol in the SC compared to the controls. The first provided a high partition into skin, but as observed in previous experiments (Table 1), does not provide a high transdermal flux, that is oleic acid favours retention of propranolol into the membrane. These results show that mechanistically, chemical enhancers and iontophoresis are completely different. While oleic acid enhanced partition of the drug into the skin, that can result in accumulation of the drug into skin iontophoresis increased the transdermal flux without facilitating drug accumulation into skin.

Overall, DPK studies with a 3-h duration agreed with the results previously obtained with much longer in vitro permeation tests (30 h) whilst allowing important time saving in screening potential transdermal formulations.

## 4. Conclusions

Pre-treatment of skin with R-(+)-limonene for 12 h and iontophoresis were the most efficient enhancement techniques for the transdermal delivery propranolol. Iontophoresis at 0.5 mA/cm^2^ was preferable in that it does not require pre-treating the skin and provided a flux 2.12-fold higher than the passive permeation and much faster delivery to the receptor solution permeation of the drug. These findings combined with the lower irritation potential of iontophoresis make this an advantageous technique to be balanced against the higher complexity of an iontophoretic device with respect a passive patch.

The DPK method was sufficiently sensitive to detect subtle vehicle-induced effects on the skin permeation of propranolol. The shorter duration of these experiments and their ability to provide mechanistic information through *KH* and *D*/*H^2^* configurate them as practical and potentially insightful approach to quantify and, ultimately, optimize topical bioavailability.

## Figures and Tables

**Table 1:** Permeation parameters of propranolol across pig ear skin: amount of drug recovered from the skin (*Q_skin_*); transdermal flux (*J*); enhancement ratio (*ER_flux_*); drug’s partition parameter (*KH*), diffusivity parameter (*D*/*H^2^*) and lag time (*t_o_*) calculated for all the chemical enhancers studied and iontophoresis conditions (mean ± SD).

Experimental Condition Assayed (*n*)	*Q*_skin_ [µg/(cm^2^)]	*J* [µg/(cm^2^h)]	*ER*_flux_	*KH* (cm)	*D*/*H*^2^ (h^−1^)	*t*_o_ (h)
Buffer (pH 7.4) [control] (12)	302 ± 81	48.9 ± 1.3	-	0.31 ± 0.03	0.026 ± 0.002	6.41 ± 0.49
Ethanol [vehicle control] (10)	261 ± 27	51.0 ± 1.2	-	0.42 ± 0.03	0.020 ± 0.001	8.13 ± 0.43
Laurocapram (6)	275 ± 19	99.7 ± 2.0 ^a,b,^*	1.74 ± 0.70	0.48 ± 0.04 ^a,^*	0.037 ± 0.003 ^a,b,^*	4.57 ± 0.38
R-(+)-limonene (6)	401 ± 53	102 ± 2 ^a,b,^*	1.88 ± 0.39	0.46 ± 0.03 ^a,^*	0.092 ± 0.01 ^a,b,^**	1.80 ± 0.27 ^a,b,^**
Oleic acid (6)	516 ± 69 ^a,b,^*	79.6 ± 2.4 ^a,b,^*	1.39 ± 0.36	0.58 ± 0.06 ^a,b,^**	0.030 ± 0.003 ^a,b,^*	5.54 ± 0.60
Decenoic acid (6)	333 ± 97	72.7 ± 9.2	-	0.39 ± 0.03	0.027 ±0.00 1 ^b,^*	6.26 ± 0.33
Iontophoresis 0.25 mA/cm^2^ (5)	252 ± 42	71.6 ± 7.1 ^a,^*	-	-	-	0.026 ± 0.011
Iontophoresis 0.5 mA/cm^2^ (5)	262 ± 28	108 ± 10 ^a,^*	2.12 ± 0.79	-	-	0.017 ± 0.014

Upperscripts denote statistically significant differences with respect to the buffer-passive control (^a^) or to the vehicle control (ethanol) (^b^) (* *p* < 0.05; ** *p* < 0.01).

**Table 2:** Partitioning (*K*) and diffusivity (*D*/*H^2^*) parameters, *AUC* and the total amount of the drug in the SC (*A*) of propranolol obtained by the tape-stripping procedure after 3 h of in vitro permeation absorption in the different conditions assayed (mean ± SD).

Experimental Condition Assayed (n)	*K*	*D*/*H*^2^ (h^−1^)	*AUC* (mg/cm^3^)	*A* (µg/cm^2^)
Buffer (pH 7.4) [control] (6)	4.37 ± 0.62 ^a^	0.11 ± 0.03	6.05 ± 1.64	5.26 ± 1.42
Ethanol [vehicle control] (6)	2.36 ± 0.32	0.23 ± 0.06	4.32 ± 0.75	3.45 ± 0.60
Laurocapram (6)	2.12 ± 0.25	0.31 ± 0.08	3.77 ± 1.77	3.47 ± 1.63
R-(+)-limonene (6)	1.55 ± 0.21	0.47 ± 0.15 ^a^	3.44 ± 1.01	3.27 ± 0.97
Oleic acid (6)	5.73 ± 1.26 ^a^	0.29 ± 0.07	10.6 ± 2.5 ^b^	8.97 ± 2.09 ^b^
Iontophoresis 0.25 mA/cm^2^ (4)	-	-	5.48 ± 0.36	5.77 ± 0.38
Iontophoresis 0.5 mA/cm^2^ (4)	-	-	7.32 ± 0.78 ^b^	7.73 ± 0.83 ^b^

ANOVA (*p* < 0.05) indicates statistical differences respective to other chemical enhancers (^a^) and among all strategies studied (^b^).
